# Interactions Between Heavy Metal Exposure and Blood Biochemistry in an Urban Population of the Black Swan (*Cygnus atratus*) in Australia

**DOI:** 10.1007/s00244-024-01055-z

**Published:** 2024-02-21

**Authors:** Damien Nzabanita, Raoul A. Mulder, Damian C. Lettoof, Stephen Grist, Jordan O. Hampton, Jasmin Hufschmid, Dayanthi Nugegoda

**Affiliations:** 1 School of Science, Royal Melbourne Institute of Technology, Melbourne, VIC 3083 Australia; 2 School of BioSciences, Faculty of Science, The University of Melbourne, Parkville, VIC 3052 Australia; 3 Centre for Environment and Life Sciences, Commonwealth Scientific and Industrial Research Organisation (CSIRO), Floreat, WA 6014 Australia; 4 School of Molecular and Life Sciences, Curtin University, Bentley, WA 6102 Australia; 5 Melbourne Veterinary School, Faculty of Science, The University of Melbourne, Werribee, VIC 3030 Australia; 6 Harry Butler Institute, Murdoch University, Murdoch, WA 6150 Australia

## Abstract

There is growing recognition of the threat posed to wildlife by pollutants. Waterbirds are robust bioindicators of ecosystem health, and metal toxicity is a threat to these species in waterways worldwide. Urban waterbirds are likely to be at the highest risk of heavy metal exposure, but this issue has not been widely explored in Australia. Our aim was to estimate contemporary heavy metal exposure in a sedentary urban waterbird population: black swans (*Cygnus atratus*) inhabiting an inner-city wetland in one of Australia’s largest cities, Melbourne. To investigate the physiological implications of legacy heavy metal exposure in these birds, we quantified blood biochemistry profiles and examined their relationships with metal concentrations in feathers. We caught 15 swans in 2021 and took feather samples to measure the concentration of eight heavy metals (chromium (Cr), manganese (Mn), iron (Fe), nickel (Ni), copper (Cu), zinc (Zn), lead (Pb), and mercury (Hg)), and blood samples to measure the concentration of 13 plasma analytes. Multivariate regression analysis revealed few associations between heavy metals and biochemistry markers, and no differences between sexes or age classes. This study presents a baseline dataset of these contaminants and blood biochemical profiles of swans at this wetland that can be used for future monitoring and is an important step toward a better understanding of the threat posed by heavy metals to Australian urban waterbirds.

**Supplementary Information:**

The online version contains supplementary material available at 10.1007/s00244-024-01055-z.

With the accelerating pace of the Anthropocene, negative impacts from chemical pollution on animals (including humans), plants, microbes and ecosystems are escalating globally. In recognition of the profound threat faced by these chemicals, the United Nations has recently declared pollution to be the third global catastrophe (along with climate change and biodiversity loss) (United Nations 2021). Chemical toxicants are of concern in aquatic systems due to their ability to spread through ecosystems and expose aquatic and terrestrial biota causing detrimental effects on health and survival. Over time, contaminants are found in increasing concentrations in waterways, discharged from industrial effluents, agricultural and urban discharge, wastewater, and stormwater runoff (Bradl 2005; Walker et al. 2012). Many such chemicals persist, bioaccumulate, and bio-magnify up food webs, with one of the most famous global examples being the near-extinction of bald eagles (*Haliaeetus leucocephalus*) due to DDT exposure, and their subsequent recovery once the chemical was banned (Grier 1982).

Waterbirds provide invaluable ‘ecosystem services’ in freshwater systems, including as vectors of seeds, invertebrates and nutrients, and acting as both predators and herbivores (Green and Elmberg 2014). They are also regarded as useful bioindicators (Amat and Green 2010), due to their frequent position in food webs as top trophic-level foragers (Andrade et al. 2018). Exposure to heavy metals can cause mortality in swans and other waterbirds (Degernes et al. 2006), as well as a variety of harmful non-lethal physiological impacts (Meissner et al. 2020). Therefore, understanding the impacts of anthropogenic toxicants on these species is crucial for their conservation and for the health of the ecosystems they inhabit. To effectively conserve freshwater bird species in the Anthropocene, it is important to identify the main pollution activities affecting each species, the sources of these pollutants, and their impacts on individual animals and population persistence (Green et al. 2017).

Since the industrial revolution, the abundance of many environmental contaminants, including heavy metals, has substantially increased in ecosystems due to urban and industrial activities (Sayadi et al. 2010). As the global human population grows and anthropogenic activity intensifies in cities, urban birds are especially susceptible to exposure to chemical contaminants, including heavy metals. In Australia, there is little information available on heavy metal exposure in waterbirds, with the exception of lead levels studied while lead shot was used for waterfowl hunting (Harper and Hindmarsh 1990; Koh and Harper 1988; Wickson et al. 1992). However, recently Szabo et al. (2022) reported elevated fecal and plasma concentrations of per- and poly-fluoroalkyl substances (PFAS), a class of persistent organic pollutant (POPs), in waterbirds caught in inner Melbourne, consistent with a highly contaminated ecosystem.

Feathers are an especially useful tissue for monitoring metal exposure because they are easy to collect and store, can be sampled from the same bird in successive years, and exact minimal harm on the individual (Golden et al. 2003). Blood (serum or plasma) biochemistry panels are very commonly used diagnostic tools in veterinary medicine to broadly gauge the health status of multiple organ systems (Harris 2009; Nie et al. 2020; O'Halloran et al. 1988). Blood biochemistry parameters have been studied, and reference ranges established, for some swan species, including mute swans (*Cygnus olor*) (O'Halloran et al. 1988), black-necked swans (*C. melanocoryphus*) (Artacho et al. 2007), and tundra swans (*C. columbianus*) (Milani et al. 2012). However, to our knowledge, no such data have been published for the only swan species found in Australia, the black swan (*C. atratus*), although published studies have reported plasma melatonin (Aulsebrook et al. 2020) and cortisol (Payne et al. 2012) levels in this species.

Here, we report results from a study on heavy metal exposure by Australian black swans residing in a highly urbanized wetland. We used feathers to quantify heavy metal body burdens and analyzed the correlations between these data and blood biochemistry parameters from the same birds. The objectives were therefore to: (a) measure heavy metal concentrations in the feathers of urban black swans, (b) measure plasma biochemical values in the same birds, and (c) explore correlations between the two sets of variables.

## Materials and Methods

### Study Species

Black swans are one of the most common waterbird species found in Australian wetlands, rivers, and urban parks. They are one of Australia’s largest waterbirds, with an average mass of 7.0 kg for males and 5.6 kg for females (Kraaijeveld et al. 2004). Black swans are relatively long-lived, with banding studies revealing that birds live for > 14 years (Williams 1973). They are socially monogamous (Kraaijeveld et al. 2004), and almost entirely herbivorous, with their diet primarily consisting of the leaves and shoots of submerged aquatic plants and algae (Smith et al. 2012). Black swans are mostly diurnal but exhibit some flexibility and will move between water bodies at night, with urban populations often displaying sedentary tendencies with established resident birds (Payne et al. 2012). Their size and tendency to habituate to humans mean that they can be captured and recaptured relatively easily (Porter et al. 2022), allowing repeated sampling, and making them ideal subjects for ecotoxicology research (Szabo et al. 2022).

### Study Site and Sampling

The study was conducted at Albert Park Lake, 3 km from the central business district (CBD) of the city of Melbourne, Australia. Melbourne is the second-largest city in Australia, with a population of around 5 million. Albert Park Lake is an artificial water body of approximately 45 hectares. The lake is home to a resident population of black swans, and this population has been extensively studied for over a decade (Guay and Mulder 2009). As a result, a large number of swans on the lake (including all individuals that participated in this study) are tagged with numbered neck collars, allowing them to be individually recognized (Guay and Mulder 2009). The Albert Park Lake population is largely resident, with substrate sources in and around the Melbourne CBD being their primary exposure sources for environmental chemicals (Szabo et al. 2022). The lake is also home to several other waterbird species (Dear et al. 2015; Payne et al. 2012).

Fifteen black swans (seven juveniles, eight adults; four females, six males, five unknown sex) were captured by hand (Douglas et al. 2022) in 2021. Samples for this study were collected under an Animal Ethics Committee (AEC) license from the University of Melbourne AEC: number 2021-10172-15083-4 (‘Urban ecology of black swans’). All work was conducted in accordance with the Prevention of Cruelty to Animals Act 1986 and associated Regulations, and the Australian Code for the Care and Use of Animals for Scientific Purposes (National Health and Medical Research Council 2013). To minimize negative animal welfare impacts during collection of the samples, we followed contemporary protocols recommended for this species (Porter et al. 2022), and restricted sample collection and duration of handling to that necessary to answer our core research questions. Briefly, we trussed the feet and wings of swans, and collected 1 mL of blood from the brachial (wing) vein, as described by Payne et al. (2012) and Aulsebrook et al. (2020). After blood sampling, five breast feathers from the same bird were plucked and placed in labeled plastic bags. After sample collection from each bird, they were immediately released.

Blood samples were immediately stored in tubes containing ethylenediaminetetraacetic acid (EDTA) and kept frozen on ice packs before being transported to a veterinary pathology laboratory. Once in the laboratory, the whole blood was centrifuged at 5000 revolutions per minute (rpm) for 20 min until plasma and blood cells were separated, and then plasma was stored at −20 °C until analyzed. Feather samples were kept in labeled plastic bags until analysis.

### Heavy Metals and Blood Biochemistry Analysis

We measured the concentration of eight heavy metals in feather samples: chromium (Cr), copper (Cu), iron (Fe), manganese (Mn), mercury (Hg), nickel (Ni), lead (Pb), and zinc (Zn). To eliminate external contamination (Aloupi et al. 2020), the feathers were washed through submersion in a 2:1 solution of chloroform (CHCl_3_) and methanol (CH_3_OH) in an orbital shaker for 24 h. Samples were then rinsed three times in deionized water and allowed to air dry for 6 h. Samples were then digested.

Once fully dry, feathers were cut as close to the rachis (shaft) as possible, weighed and placed into acid-washed (5% nitric acid (HNO_3_)) and labeled test tubes. In a fume hood, 0.5 mL of 70% nitric acid was added to each test tube and covered with glass beads on top of the test tubes to prevent sublimation and evaporation. Samples were kept at 70 °C in a block heater for 8 h. The resultant solution was allowed to cool to room temperature before dilution with 7% nitric acid and deionized water.

We then analyzed samples in a 7700® Series ICP-MS unit (Agilent Technologies, Santa Clara, USA), as per Nzabanita et al. (2023a). For quality control, trace elements in human hair ERMDB001 and Mussel-2976 standard reference materials (National Institute of Standards and Technology (NIST), Gaithersburg, USA) were both triplicated and used to determine the experimental recovery efficiency. All feather samples were run in duplicate, with mean values calculated for each sampled animal. The limits of detection and quantitation for each metal are reported in Online Appendix 1. Metal concentrations were measured as mg/kg dry weight.

For blood biochemistry, plasma samples were sent to the Clinical Pathology laboratory at the Melbourne Veterinary School. The following 13 standard blood biochemistry parameters were quantified using a Roche Cobas® Integra 400 Plus Randox Series Bi 3863 (Roche, Basel, Switzerland): urea, glucose, cholesterol, glutamate dehydrogenase (GLDH), amylase, aspartate transferase (AST), total protein, albumin, creatine kinase (CK), globulin, total bile acids, triglycerides, and uric acid.

### Statistical Analyses

**Fig. 1:**
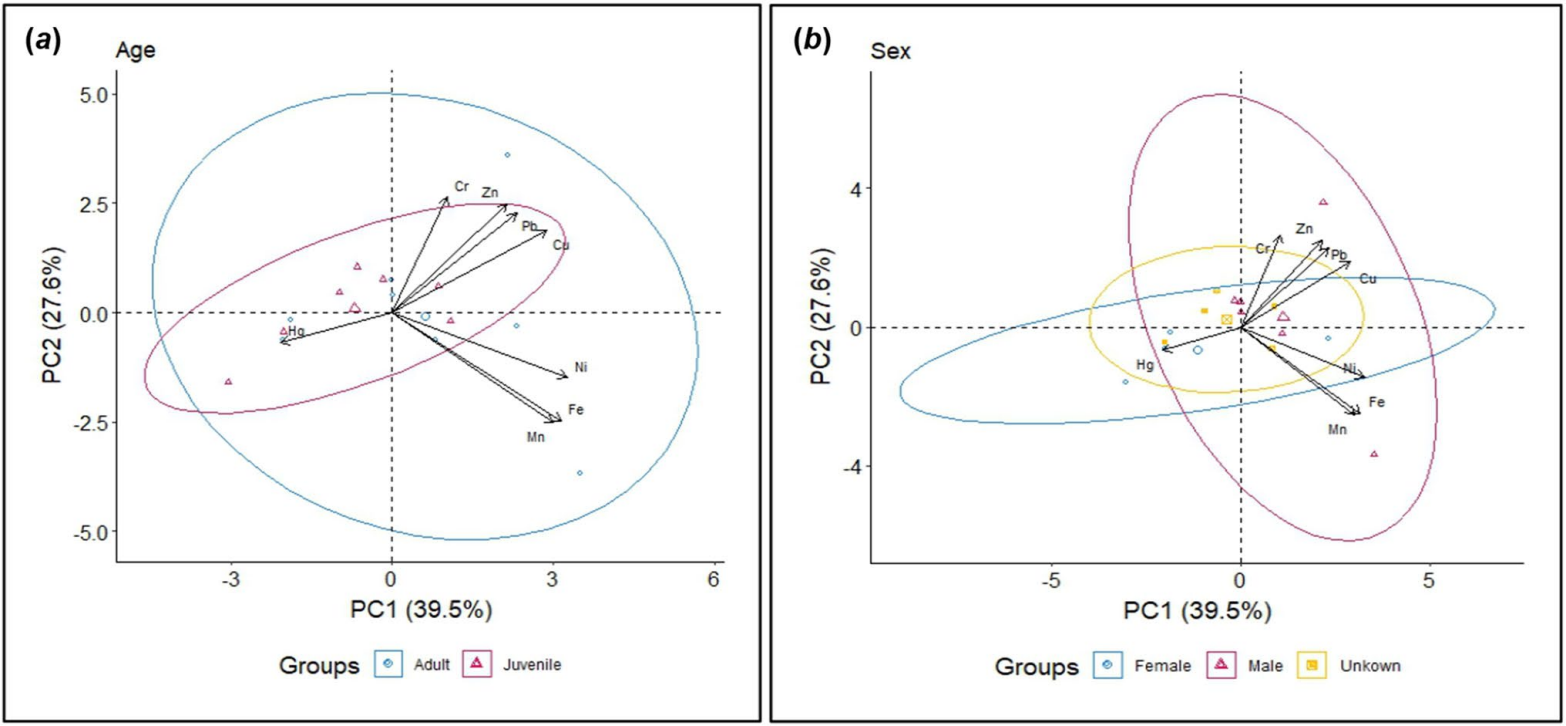
Principal Component Analysis (PCA) showing relationships between feather heavy metal concentrations, plus the effects of age (**a**) and sex (**b**), in urban black swans (*Cygnus atratus*) sampled from Albert Park Lake, Melbourne, Australia, in 2021

**Fig. 2:**
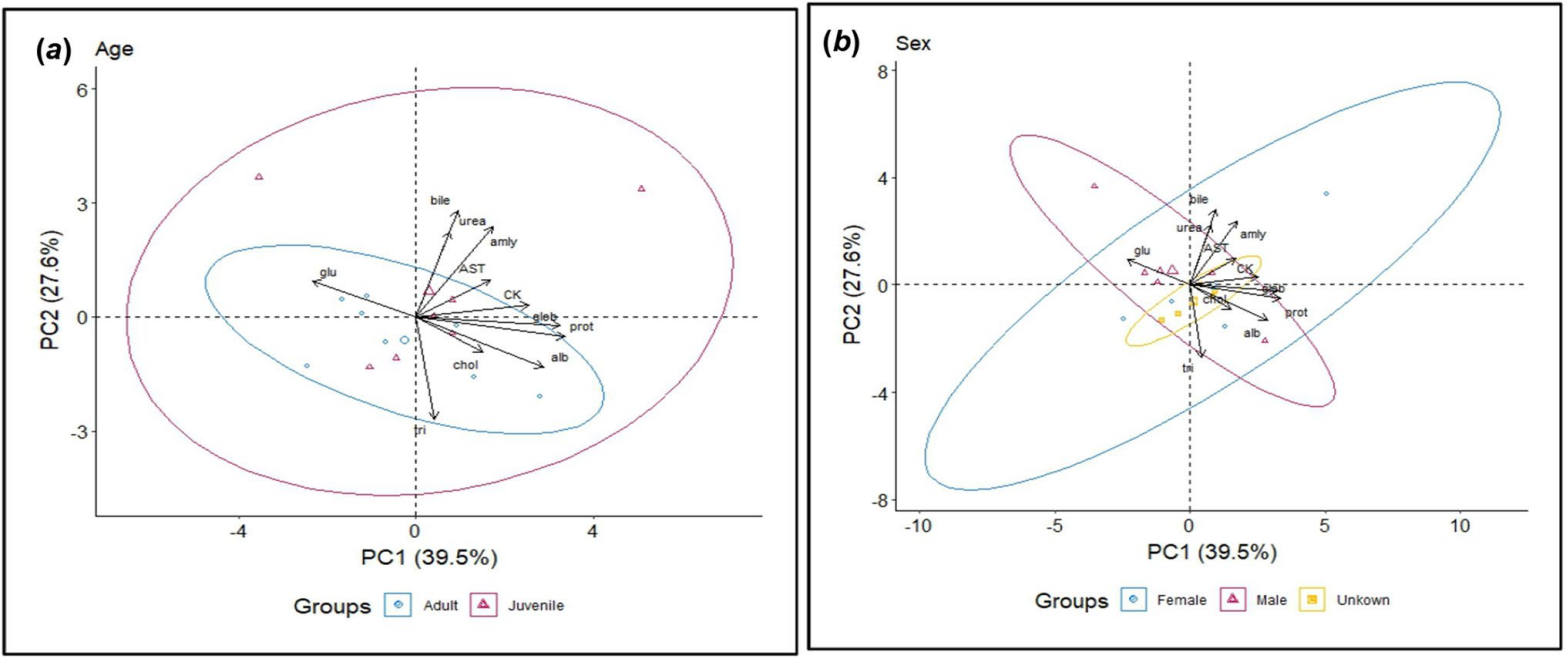
Principal Component Analysis (PCA) showing relationships between plasma biochemistry markers, plus the effects of age (**a**) and sex (**b**), in urban black swans (*Cygnus atratus*) sampled from Albert Park Lake, Melbourne, Australia, in 2021

For exploratory purposes, we performed a principal component analysis (PCA) to look for differences in heavy metal profiles, and blood analyte profiles between the age (adult or juvenile) and sex (female, male or unknown) of swans; there was substantial overlap between all groups (Figs. 1 and 2). To further identify if age or sex had an influence on metal concentrations or blood analytes, we ran a generalized linear model (GLM) with age or sex as predictors and metal or analyte concentrations as the response (log-transformed for normality where necessary). There was no significant difference between any metal (*p* > 0.12) or analyte (*p* > 0.21) and age or sex, so these were not included as covariates in subsequent models. We then ran single GLMs fitted with each metal predicting each analyte, log-transforming some metals if they made a better fit of residuals.

We adapted our statistical methods based on comparable studies that have analyzed similarly small sample sizes, and attempted comparisons between blood biochemistry variables and contaminant exposure, e.g., *n* = 16 for Malay civets (*Viverra tangalunga*) captured in Borneo (Evans et al. 2022). To deal with the analytes having missing data, we decided to either remove those samples without data for correlations (GLDH and UA: 9 and 8 birds missing data, respectively) or generate the median value for the analyte based on the other samples (amylase, AST and total bile acids; where we had values for ≥ 10 birds). All statistics were conducted in RStudio using the FactoMineR and lme4 packages (Bates 2010; Dear et al. 2015; Lê et al. 2008).

## Results

### Heavy Metals Levels

Results for heavy metal levels in feathers are shown in Table 1. The sample size for all elements except for mercury was 15, while only 4 samples returned reliable mercury concentration values. The relatively low detection rate for mercury relates to the high volatility of the element, and the potential for loss of analyte and poor recoveries after digestion (McCurdy 2011). Nonetheless, through following contemporary protocols designed to maximize mercury recovery (McCurdy 2011), we have confidence that the mercury data produced were representative of the animals we sampled for this element. The concentrations of the essential metals, zinc and iron, were the highest of the measured metals. Principal components analysis showed that there were no clear groupings between feather heavy metals versus age class (Fig. 1a) or sex (Fig. 1b). Further details are summarized in Table 1.

**Table 1:** Concentrations (mean ± SE, mg/kg) of heavy metals in feathers of 15 black swans (*Cygnus atratus*) captured at Albert Park Lake, Melbourne, Australia, in 2021

Metal	Age class		Sex		
	Juvenile (*n* = 7)	Adult (*n* = 8)	Female (*n* = 4)	Male (n = 6)	Unknown (*n* = 5)
Cr	0.16 ± 0.02	0.18 ± 0.04	0.16 ± 0.01	0.19 ± 0.05	0.15 ± 0.02
Mn	2.41 ± 0.63	4.17 ± 1.49	3.54 ± 1.65	4.42 ± 1.72	1.91 ± 0.51
Fe	45.08 ± 8.50	98.65 ± 30.34	61.14 ± 24.38	101.48 ± 37.15	50.27 ± 15.42
Hg	0.76 ± 0.22	0.27 ± 0.11	0.75 ± 0.23	0.28 ± 0.12	ND
Ni	0.24 ± 0.07	0.39 ± 0.08	0.22 ± 0.05	0.42 ± 0.08	0.27 ± 0.12
Cu	11.74 ± 0.99	11.97 ± 0.90	9.76 ± 1.64	13.24 ± 0.56	11.89 ± 0.82
Zn	133.24 ± 7.80	136.82 ± 5.76	124.02 ± 10.00	143.60 ± 6.40	133.90 ± 6.79
Pb	0.99 ± 0.15	1.54 ± 0.39	0.84 ± 0.31	1.75 ± 0.45	1.08 ± 0.22

### Plasma Biochemistry Values

Blood plasma biochemistry values are shown in Table 2. Sample sizes vary for each biochemical marker due to assay reliability. Principal components analysis showed that there were no clear groupings between plasma analytes and age (Fig. 2a) or sex (Fig. 2b). More details are summarized in Table 2.

**Table 2:** Concentrations of plasma biochemical markers (mean ± SE) in different age and sex classes from 15 wild black swans (*Cygnus atratus*) captured at Albert Park Lake, Melbourne, Australia, in 2021. Sample sizes vary for each biochemical marker due to assay reliability

Parameter	Age class		Sex		
	Adult	Juvenile	Female	Male	Unknown
Urea (mmol/L)	0.45 ± 0.04 (*n* = 8)	0.50 ± 0.07 (*n* = 7)	0.45 ± 0.08 (*n* = 4)	0.55 ± 0.04 (*n* = 6)	0.40 ± 0.08 (*n* = 5)
Glucose (mmol/L)	8.35 ± 0.44 (*n* = 8)	8.49 ± 0.73 (*n* = 7)	8.50 ± 0.74 (*n* = 4)	8.77 ± 0.77 (*n* = 6)	7.92 ± 0.51 (*n* = 5)
Cholesterol (mmol/L)	1.28 ± 0.08 (*n* = 8)	1.49 ± 0.19 (*n* = 7)	1.38 ± 0.17 (*n* = 4)	1.23 ± 0.10 (*n* = 6)	1.54 ± 0.22 (*n* = 5)
GLDH (U/L)	7.65 ± 1.47 (*n* = 4)	100.90 ± 65.27 (*n* = 2)	98.25 ± 67.14 (*n* = 2)	8.98 ± 0.77 (*n* = 4)	*N*D
Amylase (U/L)	45.00 ± 15.41 (*n* = 5)	196.83 ± 83.39 (*n* = 6)	230.00 ± 155.55 (*n* = 3)	107.00 ± 36.39 (*n* = 5)	60.33 ± 48.45 (*n* = 3)
AST (U/L)	56.88 ± 9.09 (*n* = 8)	71.17 ± 12.86 (*n* = 6)	72.50 ± 13.20 (*n* = 4)	47.50 ± 8.79 (*n* = 6)	76.75 ± 15.42 (*n* = 5)
Total protein (g/L)	35.88 ± 2.26 (*n* = 8)	35.43 ± 2.83 (*n* = 7)	38.25 ± 3.76 (*n* = 4)	33.67 ± 3.20 (*n* = 6)	36.00 ± 1.70 (*n* = 5)
Albumin (g/L)	13.00 ± 0.71 (*n* = 8)	12.71 ± 0.80 (*n* = 7)	13.5 ± 1.03 (*n* = 4)	12.33 ± 1.07 (*n* = 6)	13.00 ± 0.28 (*n* = 5)
CK (U/L)	296.88 ± 43.62 (*n* = 8)	355.86 ± 49.51 (*n* = 7)	378.75 ± 76.92 (*n* = 4)	302.50 ± 45.92 (*n* = 6)	307.2 ± 52.21 (*n* = 5)
Globulin (g/L)	22.89 ± 1.63 (*n* = 8)	22.57 ± 2.15 (*n* = 7)	24.68 ± 2.80 (*n* = 4)	21.37 ± 2.20 (*n* = 6)	22.84 ± 1.66 (*n* = 5)
Total bile acids (µmol/L)	25.21 ± 4.94 (*n* = 7)	44.39 ± 8.79 (*n* = 6)	33.74 ± 14.67 (*n* = 4)	38.59 ± 6.48 (*n* = 5)	28.73 ± 5.35 (*n* = 4)
Triglycerides (mmol/L)	0.73 ± 0.06 (*n* = 8)	0.63 ± 0.07 (*n* = 7)	0.75 ± 0.0.07 (*n* = 4)	0.62 ± 0.08 (*n* = 6)	0.70 ± 0.07 (*n* = 5)
Uric acid (µmol/L)	461.25 ± 37.02 (*n* = 4)	504.00 ± 18.70 (*n* = 3)	449.00 ± 62.23 (*n* = 2)	513.0 ± 27.35 (*n* = 3)	460.0 ± 0.00 (*n* = 2)

### Associations Between Heavy Metals and Plasma Biochemistry Profiles

There was only significant association found between feather heavy metals and plasma analytes. This was a negative relationship between cholesterol and zinc (Estimate = −0.01, *X*^2^ = 5.49, d.f. = 1, *p* = 0.02). A negative correlation between zinc and cholesterol has been reported in other bird species (Dean et al. 1991; Kucuk et al. 2003; Parak and Strakova 2011; Shah et al. 2020). However, this observation is not consistent, with other studies reporting *positive* correlations between zinc and cholesterol in other bird species (Al-Daraji and Amen 2011; Kaya et al. 2001). Regardless, at the time of writing this paper, we are unaware of any studies that clearly explain biological interactions between cholesterol and zinc in waterbirds or any vertebrate species. Hence, it is unknown whether the interactions of zinc and cholesterol at the concentrations observed in this study are clinically significant.

## Discussion

### Expectations for Heavy Metal Exposure at an Urban Site

None of the heavy metals examined in feathers were markedly elevated. The commonly cited level of concern for lead in feathers is 4 mg/kg, while for mercury, it is 5 mg/kg (Burger et al. 2009). As summarized in Table 3, the lead concentrations found in this study are similar to those reported from Victorian black swans approximately three decades ago (Wickson et al. 1992). This is an interesting result, given that the Melbourne Grand Prix (along with other motorsport events) has been held annually on a road encircling the Albert Park Lake for the last 27 years (Szabo et al. 2022). For around two weeks of every year, the lake is likely to receive the fallout of contaminants from the racing cars, although these pollutants are more likely to include ultrafine particulates, black carbon, oxides of nitrogen, and carbon monoxide than heavy metals (Brugge et al. 2007). Szabo et al. (2022) detected elevated PFAS levels in this population of black swans and suggested that this might be attributable to the aforementioned motorsport events. While the study of Szabo et al. (2022) investigated PFAS, body burdens of other classes of POPs (e.g., organochlorine pesticides (OCPs), polychlorinated bi-phenyls (PCBs), and polycyclic aromatic hydrocarbons (PAHs)) (Nzabanita et al. 2023b) have not been investigated in this species. It would be of interest to investigate their occurrence and relationship with the same plasma biochemistry parameters we measured.

**Table 3:** The concentration of heavy metals (mg/kg dry weight: mean ± SE) in feathers of swans (*Cygnus* spp.) from different global regions as reported in the published literature

Species	Country	*n*	Cr	Mn	Fe	Ni	Cu	Zn	Pb	Hg	Citation
Black swan (*Cygnus atratus*)	Australia	15	0.17 ± 0.02	3.35 ± 0.88	73.65 ± 18.03	0.32 ± 0.06	11.86 ± 0.67	135.15 ± 4.79	1.28 ± 0.23	0.52 ± 0.17	This study
Black swan	Australia	10	NR	NR	NR	NR	NR	NR	1.2 ± 0.8	0.52	(Wickson et al. 1992)
Mute swan (*C. olor*)	Hungary	17	1.02 ± 0.90	NR	NR	NR	10.24 ± 2.25	NR	1.11 ± 1.23	< 0.5	(Grúz et al. 2015)
Whooper swan (*C. cygnus*)	China	6	6.57 ± 0.59	NR	NR	NR	21.96 ± 5.33	103.49 ± 3.29	3.64 ± 1.13	0.20 ± 0.01	(Wang et al. 2017)

NR = not reported

### Comparison to other Swan Species

Some comparisons were possible with other swan species studied on other continents (Table 3). Generally, the concentrations of heavy metals we report in these black swans are lower than those in other swan species (Table 3), except for zinc and mercury, which were higher. The feather lead concentrations found in this study were similar to those reported in mute swan feathers from a freshwater lake in Hungary in 2015 (Grúz et al. 2015), and lower than those in whooper swan (*C. cygnus*) feathers from coastal north-eastern China (Wang et al. 2017). Copper concentrations were also more similar to those of mute swans from Hungary (Grúz et al. 2015) and lower than those from China (Wang et al. 2017). However, chromium levels detected in this study were lower than in Hungary and much lower than that of whooper swan feathers in China. Zinc is normally an essential trace metal and needed in heavy amounts by organisms and plays a significant role as a co-factor in metalloenzymes (McCall et al. 2000; Ye et al. 2020). However, excessive accumulation of this metal can also lead to adverse physiological effects (Carpenter et al. 2004; Gasaway and Buss 1972). Mercury, on the other hand, has no biological role (Martinez-Finley and Aschner 2014; Seewagen 2010; Walker et al. 2012), and elevated exposure to this element can cause negative reproductive and neurological effects (Seewagen 2010; Varian-Ramos et al. 2014; Whitney and Cristol 2018). Importantly, mercury can bioaccumulate (Finger et al. 2016), and mercury concentrations in feathers have been shown to be indicative of mercury body burdens in birds (Ahmadpour et al. 2016; Albert et al. 2019; Bottini et al. 2021).

### Implications for Bird Health

To our knowledge, this is the first investigation of heavy metal exposure in black swans Australia, since a single study focused on lead was published more than 30 years ago (Wickson et al. 1992). To date, there has been little research on heavy metal exposure in Australian waterbirds generally, with few studies exploring the physiological implications of different concentrations of harmful heavy metal body burdens. While reference ranges have not been established for plasma analytes in this species, the parameters measured in this study did not appear to lie outside typical range limits for other swan species (Martinez-Haro et al. 2011). Moreover, we found little evidence of relationships between metal concentrations and plasma biomarkers of health in our study animals. This suggests that the body burdens of trace metals we observed are below toxicity thresholds in this species. Alternatively, it may be that the sample size available at this site was not large enough to detect any subtle changes in these plasma biomarkers of health, given the individual variability in a population of mixed age and sex classes.

### Study Limitations

Several factors limited our ability to draw general conclusions from our data. Firstly, our sample size was small, and included birds of various ages, and some of unknown sex. In any study, when multiple statistical tests are run, there is always a chance of detecting a significant result by chance, i.e., a Type 1 error, or false positive. We acknowledge that our univariate analysis approach could have increased the chance of Type 1 errors, given that > 20 relationships were explored. However, given the sample size and exploratory nature of this investigation, multivariate analyses would have resulted in overfitting models, and would have been difficult to robustly interpret in biological terms, and were thus not conducted (Evans et al. 2022). With our small sample size, single paired comparisons were all that we had power for, and we expect any statistically significant (or close to) relationships to be suggestive at best and warrant a larger sample size to verify.

Second, we were not able to include environmental contaminant data from the sites in which animals were sampled, as has been employed for similar past studies, e.g., Szabo et al. (2022).

Third, direct comparison of our paired plasma and feather samples was complicated by temporal differences in the chemistry of these two tissue types. The concentration of metals in feather keratin reflects exposure at the time of feather growth (which may be months before sampling), as well as sometimes being influenced by excretion of excess metals into feathers (Vizuete et al. 2019). As a result, the use of feathers as an indicator of metal exposure requires knowledge of the period when the feather was grown, as well as molting patterns (Jaspers et al. 2004), whereas blood continually circulates through the body, meaning that concentrations of molecules in plasma samples typically reflect physiological events occurring in an animal at the time of sampling.

For this reason, metal concentrations in keratinized tissues (e.g., feather, hair) do not always correlate with organ or body levels, and the association between metal concentrations in keratin and internal exposure can be complicated (Lettoof et al. 2021). There is, however, evidence regarding the utility of metal levels in feathers as indicators of internal metal concentrations. For instance, a correlation was recently found between mercury levels in feathers and blood samples taken at the time of feather growth in song sparrows (*Melospiza melodia*) from Canada (Bottini et al. 2021). Additionally, several published studies have attempted analyses similar to ours and have used similar methods to those we adopted (Evans et al. 2022; Philpot et al. 2019). Despite the difficulties in navigating the different timelines associated with these two sample types, we were able to make some novel comparisons between blood and feather parameters.

## Conclusion

The present study is an important step toward a better understanding of the threat posed by heavy metals to Australian urban waterbirds. Results show that urban swans in Melbourne do not exhibit markedly elevated concentrations of contaminants, and few physiological implications were obvious through examination of plasma biochemistry markers. While small sample size studies such as ours suffer from a high degree of uncertainty, our data represent a useful baseline for future monitoring of other species of waterbirds that feed in similar urban wetlands. We suggest that future studies could also explore the physiological effects in black swans of other common urban toxicants, including microplastics and POPs classes other than PFAS.

### Supplementary Information

Below is the link to the electronic supplementary material.

Supplementary file1 (DOCX 41 KB)

## Data Availability

The datasets generated during and/or analyzed during the current study are available from the corresponding author on reasonable request.

## References

[CR1] Ahmadpour M, Lan-Hai L, Ahmadpour M, Hoseini SH, Mashrofeh A, Binkowski ŁJ (2016). Mercury concentration in the feathers of birds from various trophic levels in fereydunkenar international wetland (Iran). Environ Monit Assess.

[CR2] Albert C, Renedo M, Bustamante P, Fort J (2019). Using blood and feathers to investigate large-scale Hg contamination in Arctic seabirds: a review. Environ Res.

[CR3] Al-Daraji HJ, Amen MHM (2011). Effect of dietary zinc on certain blood traits of broiler breeder chickens. Int J Poult Sci.

[CR4] Aloupi M, Ferentinou E, Zaharaki O-M, Akriotis T (2020). Does dilute nitric acid improve the removal of exogenous heavy metals from feathers? A comparative study towards the optimization of the cleaning procedure of feather samples prior to metal analysis. Ecotoxicol Environ Saf.

[CR5] Amat JA, Green AJ, Hurford C, Schneider M, Cowx I (2010). Waterbirds as bioindicators of environmental conditions. Conservation Monitoring in Freshwater Habitats.

[CR6] Andrade R, Bateman HL, Franklin J, Allen D (2018). Waterbird community composition, abundance, and diversity along an urban gradient. Landsc Urban Plan.

[CR7] Artacho P, Soto-Gamboa M, Verdugo C, Nespolo RF (2007). Blood biochemistry reveals malnutrition in black-necked swans (*Cygnus melanocoryphus*) living in a conservation priority area. Comp Biochem Physiol a: Mol Integr Physiol.

[CR8] Aulsebrook AE, Lesku JA, Mulder RA, Goymann W, Vyssotski AL, Jones TM (2020). Streetlights disrupt night-time sleep in urban black swans. Front Ecol Evol.

[CR9] Bates DM (2010). Linear mixed-effects models using 'Eigen' and S4.

[CR10] Bottini CLJ, MacDougall-Shackleton SA, Branfireun BA, Hobson KA (2021). Feathers accurately reflect blood mercury at time of feather growth in a songbird. Sci Total Environ.

[CR11] Bradl HB, Bradl HB (2005). Sources and origins of heavy metals. Interface science and technology.

[CR12] Brugge D, Durant JL, Rioux C (2007). Near-highway pollutants in motor vehicle exhaust: a review of epidemiologic evidence of cardiac and pulmonary health risks. Environ Health.

[CR13] Burger J, Gochfeld M, Jeitner C (2009). Mercury and other metals in eggs and feathers of glaucous-winged gulls (*Larus glaucescens*) in the Aleutians. Environ Monit Assess.

[CR14] Carpenter JW, Andrews GA, Beyer WN (2004). Zinc toxicosis in a free-flying trumpeter swan (*Cygnus buccinator*). J Wildl Dis.

[CR15] Dean CE, Hargis BM, Hargis PS (1991). Effects of zinc toxicity on thyroid function and histology in broiler chicks. Toxicol Lett.

[CR16] Dear EJ, Guay P-J, Robinson RW, Weston MA (2015). Distance from shore positively influences alert distance in three wetland bird species. Wetlands Ecol Manage.

[CR17] Degernes L, Heilman S, Trogdon M (2006). Epidemiologic investigation of lead poisoning in trumpeter and tundra swans in Washington state, USA, 2000–2002. J Wildl Dis.

[CR18] Douglas TK, Weston MA, Greenwell CN, Smith BP, Waudby HP, Alberthsen C, Hampton JO (2022). Research methods for birds. Wildlife research in Australia: practical abd applied methods.

[CR19] Evans MN, Waller S, Müller CT (2022). The price of persistence: assessing the drivers and health implications of metal levels in indicator carnivores inhabiting an agriculturally fragmented landscape. Environ Res.

[CR20] Finger A, Lavers JL, Orbell JD, Dann P, Nugegoda D, Scarpaci C (2016). Seasonal variation and annual trends of metals and metalloids in the blood of the little penguin (*Eudyptula minor*). Mar Pollut Bull.

[CR21] Gasaway WC, Buss IO (1972). Zinc toxicity in the mallard duck. J Wildl Manag.

[CR22] Golden NH, Rattner BA, Cohen JB, Hoffman DJ, Russek-Cohen E, Ottinger MA (2003). Lead accumulation in feathers of nestling black-crowned night herons (*Nycticorax nycticorax*) experimentally treated in the field. Environ Toxicol Chem.

[CR23] Green AJ, Elmberg J (2014). Ecosystem services provided by waterbirds. Biol Rev.

[CR24] Green AJ, Alcorlo P, Peeters ETHM (2017). Creating a safe operating space for wetlands in a changing climate. Front Ecol Environ.

[CR25] Grier JW (1982). Ban of DDT and subsequent recovery of reproduction in bald eagles [*Haliaeetus leucocephalus*, Canada]. Science.

[CR26] Grúz A, Szemerédy G, Kormos É (2015). Monitoring of heavy metal burden in mute swan (*Cygnus olor*). Environ Sci Pollut Res.

[CR27] Guay P-J, Mulder R (2009). Do neck-collars affect the behaviour and condition of Black Swans (*Cygnus atratus*)?. Emu-Austr Ornithol.

[CR28] Harper M, Hindmarsh M (1990). Lead poisoning in magpie geese *Anseranas semipalmata* from ingested lead pellet at bool lagoon game reserve (South Australia). Wildl Res.

[CR29] Harris DJ, Tully TN, Dorrestein GM, Jones AK, Cooper JE (2009). Clinical tests. Handbook of avian medicine.

[CR30] Jaspers V, Dauwe T, Pinxten R (2004). The importance of exogenous contamination on heavy metal levels in bird feathers. A field experiment with free-living great tits, Parus major. J Environ Monit.

[CR31] Kaya S, Keçeci T, Haliloğlu S (2001). Effects of zinc and vitamin A supplements on plasma levels of thyroid hormones, cholesterol, glucose and egg yolk cholesterol of laying hens. Res Vet Sci.

[CR32] Koh T, Harper M (1988). Lead poisoning in black swans, *Cygnus atratus*, exposed to spent lead shot at bool lagoon game reserve. South Austr Wildl Res.

[CR33] Kraaijeveld K, Carew P, Billing T, Adcock GJ, Mulder RA (2004). Extra-pair paternity does not result in differential sexual selection in the mutually ornamented black swan (*Cygnus atratus*). Mol Ecol.

[CR34] Kucuk O, Sahin N, Sahin K (2003). Supplemental zinc and vitamin A can alleviate negative effects of heat stress in broiler chickens. Biol Trace Elem Res.

[CR35] Lê S, Josse J, Husson F (2008). FactoMineR: an R package for multivariate analysis. J Stat Softw.

[CR36] Lettoof D, Rankenburg K, McDonald B (2021). Snake scales record environmental metal (loid) contamination. Environ Pollut.

[CR37] Martinez-Finley EJ, Aschner M (2014). Recent advances in mercury research. Curr Environ Health Reports.

[CR38] Martinez-Haro M, Green AJ, Mateo R (2011). Effects of lead exposure on oxidative stress biomarkers and plasma biochemistry in waterbirds in the field. Environ Res.

[CR39] McCall KA, Huang C-c, Fierke CA (2000). Function and mechanism of zinc metalloenzymes. J Nutr.

[CR40] McCurdy E (2011) Successful low level mercury analysis using the Agilent 7700 series ICP-MS. Agilent ICP-MS Journal:45

[CR41] Meissner W, Binkowski ŁJ, Barker J, Hahn A, Trzeciak M (2020). Relationship between blood lead levels and physiological stress in mute swans (*Cygnus olor*) in municipal beaches of the southern Baltic. Sci Total Environ.

[CR42] Milani JF, Wilson H, Ziccardi M, LeFebvre R, Scott C (2012). Hematology, plasma chemistry, and bacteriology of wild tundra swans (*Cygnus columbianus*) in Alaska. J Wildl Dis.

[CR43] National Health and Medical Research Council (2013) australian code for the care and use of animals for scientific purposes. In: 8th edn. National health and medical research council, Canberra, Australia

[CR44] Nie D, Gui J, Zhao N (2020). Haematological and serum biochemical reference values in Chinese water deer (*Hydropotes inermis*): a preliminary study. BMC Vet Res.

[CR45] Nzabanita D, Hampton JO, Toop SD (2023). Expanding the use of portable XRF to monitor lead exposure in an Australian duck species two decades after a ban on lead shot. Sci Total Environ.

[CR46] Nzabanita D, Shen H, Grist S (2023). Exposure to POPs in Australian waterbirds. Environ Toxicol Chem.

[CR47] O'Halloran J, Duggan PF, Myers AA (1988). Biochemical and haematological values for mute swans (*Cygnus olor*): effects of acute lead poisoning. Avian Pathol.

[CR48] Parak T, Strakova E (2011). Zinc as a feed supplement and its impact on plasma cholesterol concentrations in breeding cocks. Acta Vet Brno.

[CR49] Payne CJ, Jessop TS, Guay P-J, Johnstone M, Feore M, Mulder RA (2012). Population, behavioural and physiological responses of an urban population of black swans to an intense annual noise event. PLoS ONE.

[CR50] Philpot SM, Lavers JL, Nugegoda D, Gilmour ME, Hutton I, Bond AL (2019). Trace element concentrations in feathers of seven petrels (*Pterodroma*
*spp*.). Environ Sci Pollut Res.

[CR51] Porter J, Spencer J, O'Neill S, Brandis KJ, Watson MJ, Smith BP, Waudby HP, Alberthsen C, Hampton JO (2022). Aquatic birds. Wildlife research in Australia: practical and applied methods.

[CR52] Sayadi MH, Sayyed MRG, Kumar S (2010). Short-term accumulative signatures of heavy metals in river bed sediments in the industrial area, Tehran. Iran Environ Monitor Assess.

[CR53] Seewagen CL (2010). Threats of environmental mercury to birds: knowledge gaps and priorities for future research. Bird Conserv Int.

[CR54] Shah M, Zaneb H, Masood S (2020). Effect of single or combined supplementation of zinc and probiotics on muscle and bone characteristics and haematobiochemical profile in broilers. Veterinární Medicína.

[CR55] Smith AN, Vernes KA, Ford HA (2012). Grazing effects of Black Swans *Cygnus atratus* (Latham) on a seasonally flooded coastal wetland of eastern Australia. Hydrobiologia.

[CR56] Szabo D, Moodie D, Green MP, Mulder RA, Clarke BO (2022). Field-based distribution and bioaccumulation factors for cyclic and aliphatic per-and polyfluoroalkyl substances (PFASs) in an urban sedentary waterbird population. Environ Sci Technol.

[CR57] United Nations (2021) What is the Triple Planetary Crisis? United Nations framework convention on climate change, New York City, USA. Report available on-line at: https://unfccc.int/blog/what-is-the-triple-planetary-crisis

[CR58] Varian-Ramos CW, Swaddle JP, Cristol DA (2014). Mercury reduces avian reproductive success and imposes selection: an experimental study with adult- or lifetime-exposure in zebra finch. PLoS ONE.

[CR59] Vizuete J, Pérez-López M, Míguez-Santiyán MP, Hernández-Moreno D (2019). Mercury (Hg), lead (Pb), cadmium (Cd), selenium (Se), and arsenic (As) in liver, kidney, and feathers of gulls: a review. Rev Environ Contam Toxicol.

[CR60] Walker CH, Sibly RM, Hopkin SP, Peakall DB (2012). Principles of ecotoxicology.

[CR61] Wang F, Xu S, Zhou Y, Wang P, Zhang X (2017). Trace element exposure of whooper swans (*Cygnus cygnus*) wintering in a marine lagoon (Swan Lake), northern China. Mar Pollut Bull.

[CR62] Whitney MC, Cristol DA, de Voogt P (2018). Impacts of sublethal mercury exposure on birds: a detailed review. Reviews of environmental contamination and toxicology.

[CR63] Wickson R, Norman F, Bacher G, Garnham J (1992). Concentrations of lead in bone and other tissues of Victorian waterfowl. Wildl Res.

[CR64] Williams M (1973). Mortality of the black swan in New Zealand-a progress report. Wildfowl.

[CR65] Ye R, Tan C, Chen B, Li R, Mao Z (2020). Zinc-containing metalloenzymes: inhibition by metal-based anticancer agents. Front Chem.

