# Supplementary material for: Interactions Between Heavy Metal Exposure and Blood Biochemistry in an Urban Population of the Black Swan (Cygnus atratus) in Australia
Source: Arch Environ Contam Toxicol. 2024 Feb 21;86(2):178–86. doi: 10.1007/s00244-024-01055-z (PMC10904524; doi:10.1007/s00244-024-01055-z)
Supplement: Supplementary file 1 — Supplementary file1 (DOCX 41 KB) [file 244_2024_1055_MOESM1_ESM.docx]

**Interactions Between Heavy Metal Exposure and Blood Biochemistry in an Urban Population of the Black Swan (*Cygnus atratus*) in Australia**

**Damien Nzabanita****^1^** **· Raoul A. Mulder^2^ · Damian C. Lettoof ^3,4^** **· Stephen Grist^1^ · Jordan O. Hampton^5,6*^ · Jasmin Hufschmid^5^ · Dayanthi Nugegoda^1^**

**Electronic Supplementary Material**

**Table 1.** Limits of detection and quantification for eight metals analyzed in feather samples from black swans (*Cygnus atratus*) in Australia in 2021.

| **Element** | **Limit of detection (mg/kg)** | **Limit of quantification (mg/kg)** |
| --- | --- | --- |
| **Cr** | **0.022** | **0.070** |
| **Cu** | **0.059** | **0.175** |
| **Fe** | **0.047** | **3.5** |
| **Hg** | **0.012** | **0.070** |
| **Mn** | **0.007** | **0.035** |
| **Ni** | **0.016** | **0.035** |
| **Pb** | **0.005** | **0.035** |
| **Zn** | **0.033** | **0.070** |
